# Development and validation of a novel AI-derived index for predicting COPD medical costs in clinical practice

**DOI:** 10.1016/j.csbj.2025.01.015

**Published:** 2025-01-27

**Authors:** Guan-Heng Liu, Chin-Ling Li, Chih-Yuan Yang, Shih-Feng Liu

**Affiliations:** a Department of Artificial Intelligence, Chang Gung University, Taoyuan 333, Taiwan; b Department of Respiratory Therapy, Kaohsiung Chang Gung Memorial Hospital, Kaohsiung 833, Taiwan; c Division of Pulmonary and Critical Care Medicine, Department of Internal Medicine, Kaohsiung Chang Gung Memorial Hospital, Kaohsiung 833, Taiwan; d Medical Department, College of Medicine, Chang Gung University, Taoyuan 333, Taiwan

**Keywords:** COPD, MCPI, Gradient boosting model, Recursive Feature Elimination, 5-fold cross-validation

## Abstract

**Background:**

Chronic Obstructive Pulmonary Disease (COPD) is a major contributor to global morbidity and healthcare costs. Accurately predicting these costs is crucial for resource allocation and patient care. This study developed and validated an AI-driven COPD Medical Cost Prediction Index (MCPI) to forecast healthcare expenses in COPD patients.

**Methods:**

A retrospective analysis of 396 COPD patients was conducted, utilizing clinical, demographic, and comorbidity data. Missing data were addressed through advanced imputation techniques to minimize bias. The final predictors included interactions such as Age × BMI, alongside Tumor Presence, Number of Comorbidities, Acute Exacerbation frequency, and the DOSE Index. A Gradient Boosting model was constructed, optimized with Recursive Feature Elimination (RFE), and evaluated using 5-fold cross-validation on an 80/20 train-test split. Model performance was assessed with Mean Squared Error (MSE), Mean Absolute Error (MAE), Mean Absolute Percentage Error (MAPE), and R-squared (R²).

**Results:**

On the training set, the model achieved an MSE of 0.049, MAE of 0.159, MAPE of 3.41 %, and R² of 0.703. On the test set, performance metrics included an MSE of 0.122, MAE of 0.258, MAPE of 5.49 %, and R² of 0.365. Tumor Presence, Age, and BMI were identified as key predictors of cost variability.

**Conclusions:**

The MCPI demonstrates strong potential for predicting healthcare costs in COPD patients and enables targeted interventions for high-risk individuals. Future research should focus on validation with multicenter datasets and the inclusion of additional socioeconomic variables to enhance model generalizability and precision.

## Graphical Abstract

**Figure:**
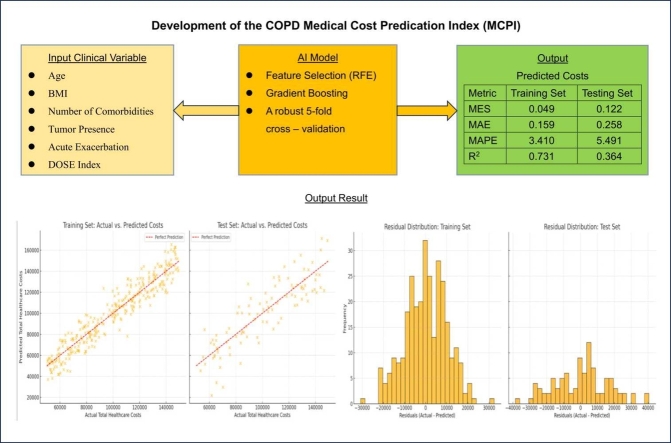

## 1 Introduction

Chronic Obstructive Pulmonary Disease (COPD) is a prevalent respiratory disorder and a leading cause of morbidity and mortality worldwide [1], [2]. The World Health Organization (WHO) projects that COPD will become the third leading cause of death globally by 2030, underscoring its significant impact on healthcare systems [3], [4]. The disease is characterized by chronic respiratory symptoms and airflow limitation [5], [6], commonly exacerbated by environmental and lifestyle factors, particularly smoking [7], [8], [9], [10]. COPD’s progression is frequently marked by acute exacerbations—sudden worsening of symptoms that often require immediate, intensive medical intervention [11], [12], [13]. These episodes are among the most significant cost drivers in COPD management, leading to increased hospital admissions, emergency visits, and resource utilization [14], [15].

Accurately predicting healthcare costs in COPD patients is essential for effective management and resource allocation. Traditional cost prediction models have generally relied on limited demographic and clinical variables, such as age, gender, and disease severity, which often fail to capture the complexity of COPD-related healthcare needs [16], [17]. Although indices like BODE (Body mass index, airflow Obstruction, Dyspnea, and Exercise capacity) and DOSE (Dyspnea, Obstruction, Smoking, Exacerbations) provide insights into disease severity and patient prognosis [18], [19], [20], they lack the specificity needed to forecast healthcare costs accurately. This limitation emphasizes the need for a more comprehensive, adaptable tool that incorporates multiple clinical dimensions relevant to COPD costs [21], [22].

Advancements in artificial intelligence (AI) and machine learning have made it possible to develop more sophisticated predictive models capable of capturing complex patterns in healthcare data [23], [24]. AI algorithms, such as Gradient Boosting models, excel in handling high-dimensional datasets, integrating multiple variables, and identifying non-linear relationships, which are essential for the nuanced cost prediction needed in COPD management [25], [26], [27], [28]. Leveraging AI can facilitate a more accurate and personalized approach to forecasting COPD-related expenses, enabling proactive risk management and cost-effective care strategies [29], [30].

In this study, we developed the COPD Medical Cost Prediction Index (MCPI), an AI-derived index aimed at predicting healthcare costs in COPD patients [31], [32]. Using a Gradient Boosting model trained on data from 396 patients, the MCPI integrates five key clinical predictors: Age × BMI, Number of Comorbidities, Tumor Presence, Acute Exacerbation, and DOSE Index. The model was optimized through grid search with cross-validation, with its predictive performance assessed using multiple metrics, including MSE, MAE, MAPE, and R² [33], [34], [35]. By identifying patients at risk for high medical costs, the MCPI holds potential as a clinical tool to inform personalized care, targeted interventions, and efficient resource allocation.

## 2 Methods

### 2.1 Study design and population

This retrospective cohort study included 396 patients diagnosed with COPD, with data collected from Chang Gung Memorial Hospital. Each record contained demographic, clinical, and comorbidity metrics relevant to predicting healthcare costs. Inclusion criteria were applied to ensure the study population was representative of COPD patients. This study developed and validated an AI-driven COPD Medical Cost Prediction Index (MCPI) to forecast healthcare expenses in COPD patients.

### 2.2 Variable selection and data processing

To minimize information loss and reduce bias, the dataset was re-analyzed using multiple imputation methods rather than excluding records with missing data. Variable selection for the COPD Medical Cost Prediction Index (MCPI) combined statistical analysis, automated feature selection, and clinical expertise. Initial Pearson correlation analysis assessed the relationships between candidate predictors and total healthcare costs, with variables having an absolute correlation coefficient (|r|) > 0.2 and p < 0.05 considered for further evaluation.

Using Recursive Feature Elimination (RFE) and clinical judgment, five key predictors were identified as significant: Age × BMI (interaction term), Number of Comorbidities, Tumor Presence, Acute Exacerbation, and the DOSE Index. Each variable was standardized using z-scores to ensure comparability. Highly collinear variables were excluded to enhance interpretability, and total healthcare costs were log-transformed to address skewness in the distribution.

### 2.3 Model construction

The MCPI was developed using a Gradient Boosting model, chosen for its ability to handle non-linear relationships and high-dimensional data. Hyperparameter tuning was conducted via grid search with cross-validation to identify the optimal values for parameters:

• Maximum tree depth: [3, 5, 7]
• Minimum samples per split: [2, 5, 10]
• Learning rate: [0.01, 0.05, 0.1]
• Number of estimators: [50, 100, 150]

To prevent overfitting, early stopping was implemented during training. The model incorporated additional temporal variables, such as seasonality and trends, to capture variations in healthcare costs over time.

### 2.4 Model validation and evaluation

The model's reliability was ensured through robust 5-fold cross-validation. Performance was evaluated using the following metrics:

• Mean Squared Error (MSE)
• Mean Absolute Error (MAE)
• Mean Absolute Percentage Error (MAPE)
• R-squared (R²)

Residual analysis was performed to identify systematic prediction errors. Feature importance was also evaluated within the Gradient Boosting model to quantify the relative contribution of each predictor. Stratified sampling was applied to ensure the training and test sets reflected the full range of cost distributions, improving model generalization.

### 2.5 Ethical considerations

All patient data were anonymized and analyzed in accordance with Chang Gung Memorial Hospital’s ethical standards and the Declaration of Helsinki. This study was approved by the hospital’s Institutional Review Board (IRB number: 201701293B0), with informed consent waived due to its retrospective nature.

## 3 Statistical analysis

All statistical analyses were performed using Python (version 3.8) and relevant libraries for data management, modeling, and statistical evaluation. The analysis followed these key steps:

• **Data Management:**

∘ The pandas library (version 1.3.3) was employed for preprocessing the dataset, addressing missing data using advanced multiple imputation techniques, and standardizing variables via z-scores to ensure consistency and comparability.

• **Model development and evaluation:**

∘ The scikit-learn library (version 0.24.2) was used to perform Recursive Feature Elimination (RFE) for variable selection, develop the Gradient Boosting model, and evaluate its performance.
∘ GridSearchCV was utilized for hyperparameter optimization, incorporating a 5-fold cross-validation strategy to identify the best parameter combinations for model performance.

• **Statistical evaluation:**

∘ The scipy library (version 1.7.1) was applied to calculate Pearson correlation coefficients and their corresponding p-values, which were used to assess the relationships between candidate predictors and total healthcare costs.

Performance metrics included Mean Squared Error (MSE), Mean Absolute Error (MAE), Mean Absolute Percentage Error (MAPE), and R-squared (R²). Statistical significance was determined using a threshold of p < 0.05.

## 4 Results

### 4.1 Patient characteristics

**Fig. 1:**
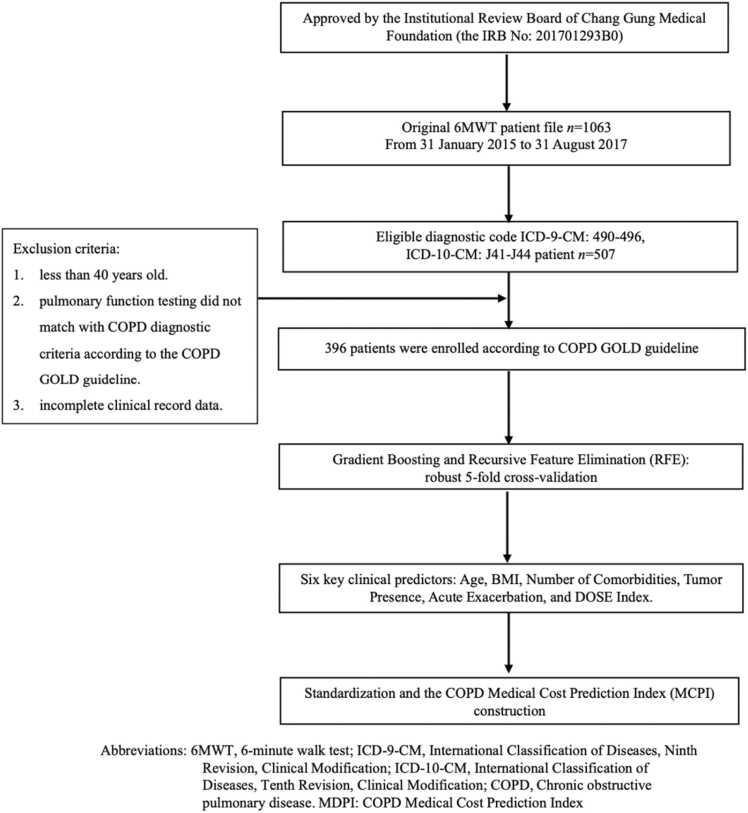
Flow chart of selected participants and identification of key variables for COPD Medical Cost Prediction Index.

The dataset comprised 396 COPD patients, whose demographics, clinical characteristics, and associated medical costs were analyzed to provide a comprehensive population overview. Table 1 summarizes the key characteristics. The average age of the patients was 73.1 years (± 9.5), with 96.5 % being male. Patients reported a mean smoking history of 31.7 pack-years (± 18.5) and an average BMI of 23.5 (± 4.1). Lung function tests showed a mean FEV1 of 55.2 % (± 18.2) of the predicted value, with 47.2 % of patients in the moderate GOLD stage. The average DLCO was 68.5 % (± 21.0), and the mean mMRC dyspnea score was 1.72 (± 0.9). The average distance walked during the 6-minute walking test was 351.9 m (± 111.6). The BODE, ADO, and DOSE indices had respective mean values of 3.0 (± 2.1), 4.9 (± 1.8), and 2.6 (± 1.1). On average, patients experienced 0.27 (± 0.75) acute exacerbations annually, with 37 % having a malignant tumor. The mean number of comorbidities was 1.27 (± 0.89), and the total medical costs averaged 750,000 NTD (± 350,000 NTD) (Table 1). Fig. 1

**Table 1:** Baseline characteristics of Chronic Obstructive Pulmonary Disease (COPD) patients.

**Factors**	**Mean ± Standard Deviation (SD) or*****n*****(%)*****n = 396***
Age (years)	73.1 ± 9.5
Male (%)	382 (96.5)
Body-mass index (BMI)	23.5 ± 4.1
Smoking	
Yes No	347(87.6 %) 49(12.4 %)
Smoking history (pack-years)	31.7 ± 18.5
FVC (% of predicted value)	79.7 ± 16.7
FEV1 (% of predicted value)	55.2 ± 18.2
FEV1/FVC (%)	52.7 ± 10.6
GOLD stage (%)	
Mild (I)	46 (11.6)
Moderate (II)	187 (47.2)
Severe (III)	140 (35.4)
Very severe (IV)	23 (5.8)
DLCO (%)	68.5 ± 21.0
6-MWD (m)	351.9 ± 111.6
MIP	72.2 ± 30.5
MEP	98.3 ± 46.8
mMRC dyspnea scale	
Scale 0/1/2/3/4	25/133/173/56/9
Exacerbations in previous year	
0–1 2–3 > 3	370(93.4 %) 21(5.3 %) 5(1.3 %)
CCI	3.3 ± 2.8
BODE INDEX	3.0 ± 2.1
ADO INDEX	4.9 ± 1.8
DOSE INDEX	2.6 ± 1.1
BODE quartile: Q1, Q2, Q3, Q4(%)	
quartile 1	188 (47.5)
quartile 2	109 (27.5)
quartile 3	71 (17.9)
quartile 4	28 (7.1)
ADO quartile: Q1, Q2, Q3, Q4 (%)	
quartile 1	40 (10.1)
quartile 2	124(31.3)
quartile 3	152 (38.4)
quartile 4	80 (20.2)
DOSE quartile: Q1, Q2, Q3, Q4 (%)	
quartile 1	214 (54 %)
quartile 2	164 (41.4 %)
quartile 3	18 (4.5 %)
quartile 4	0 (0 %)
Number of Comorbidities	1.27 ± 0.89
Acute Exacerbation	0.27 ± 0.75
Malignant Tumor	0.37 ± 0.98
Total Medical Costs (NTD)	750,000 ± 350,000

*Quartile 1 was defined by a score of 0–2, quartile 2 by a score of 3–4, quartile 3 by a score of 5–6, and quartile 4 by a score of 7–10. Abbreviations: FVC, forced vital capacity; FEV1, forced expiratory volume in 1 s; 6 MWD, 6-min walking distance; MRC score, Medical Research Council dyspnoea scale; GOLD, Global Initiative for Chronic Obstructive Lung Disease; CCI, Charlson comorbidity index; DOSE index, composite index of dyspnea, airflow obstruction, smoking status, and exacerbation frequency; BODE index, composite index of body mass index, airflow maximum expiratory pressure obstruction, dyspnoea, and exercise capacity; ADO index, composite index of age, dyspnoea, and airflow obstruction.

### 4.2 Feature importance in MCPI model

**Fig. 2:**
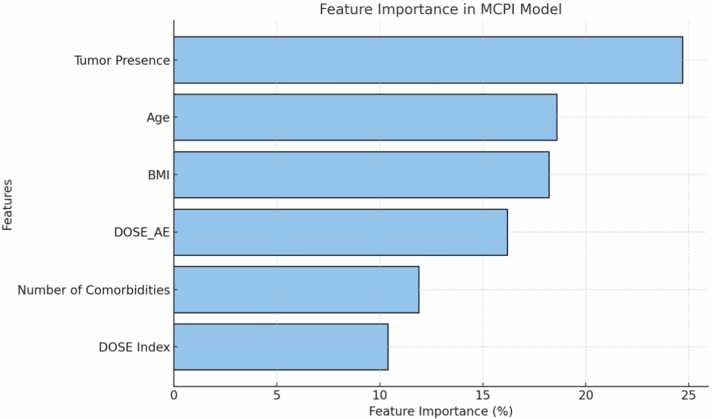
Feature importance analysis in the Gradient Boosting model revealed the relative contribution of each predictor to the prediction of COPD medical costs.

Feature importance analysis in the Gradient Boosting model revealed the relative contribution of each predictor to the prediction of medical costs. Malignant tumor presence (24.7 %) was identified as the most significant variable, followed by age (18.6 %) and BMI (18.2 %), indicating their substantial influence on total healthcare expenditures. The remaining predictors— Acute Exacerbation (16.2 %), number of Comorbidities (11.9 %), and DOSE Index (10.4 %)—also played critical roles in determining costs but to a lesser extent (Fig. 2).

The importance rankings provide valuable insights into the factors driving medical expenses in COPD patients, emphasizing the need for targeted interventions for those with malignancy, advanced age, or higher BMI. The visualization of feature importance helps clinicians understand the relative weight of each variable, supporting informed decision-making and personalized care strategies. Table 2

**Table 2:** Performance Metrics for MCPI Model.

Gradient Boosting Model Performance Metrics			
	Metric	Training Set	Testing Set
1	MES	0.049094065165904706	0.12231730926948356
2	MAE	0.15916157996668065	0.25830001799941504
3	MAPE	3.410242861120933	5.491587919030563
4	R-squared	0.7031625529887463	0.3645556959753883

### 4.3 Model performance

The COPD Medical Cost Prediction Index (MCPI) model demonstrated robust predictive capability, explaining 70.3 % of the variance in total medical costs for the training set (R² = 0.703) and 36.5 % for the test set (R² = 0.365). Key performance metrics are summarized as follows:

• Training Set:

∘ Mean Squared Error (MSE): 0.049
∘ Mean Absolute Error (MAE): 0.159
∘ Mean Absolute Percentage Error (MAPE): 3.41 %

• Test Set:

∘ Mean Squared Error (MSE): 0.122
∘ Mean Absolute Error (MAE): 0.258
∘ Mean Absolute Percentage Error (MAPE): 5.49 %

These results highlight the model's strong performance in the training set and its reasonable generalization to the test set. However, the discrepancy between training and test results suggests opportunities for further refinement, such as reducing overfitting or improving feature representation.

### 4.4 Visualization of model performance

**Fig. 3:**
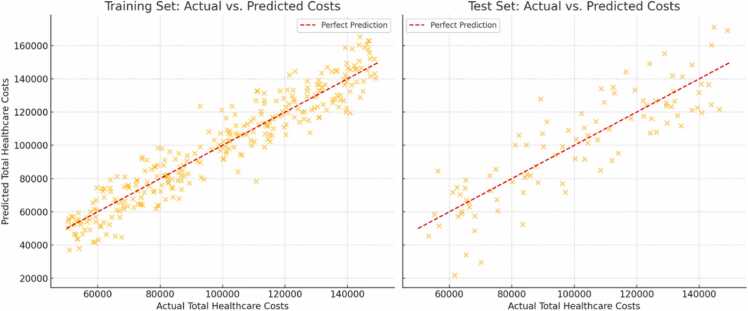
The scatter plot compares actual costs and the predicted costs in the training set and test set. The red dashed line represents perfect prediction (actual = predicted).

**Fig. 4:**
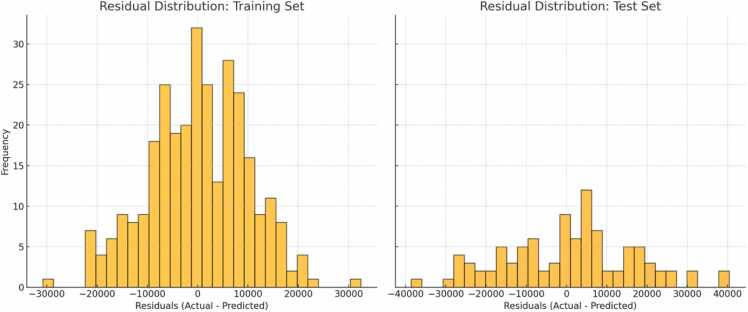
Residual Distribution Plots shows the distribution of residuals (differences between actual and predicted costs) in the training set and test set.

Fig. 3, Fig. 4 illustrate the model’s predictive performance. The scatter plot (Fig. 3) compares actual costs and the predicted costs in the training set and test set. The red dashed line represents perfect prediction (actual = predicted). The high alignment with the perfect prediction line also reflects the model's low training error, consistent with the performance metrics (e.g., low MSE and high R² for the training set). In the test set scatter plot, points still align with the red dashed line, but there is more scatter compared to the training set. Residual Distribution Plots (Fig. 4) shows the distribution of residuals (differences between actual and predicted costs) in the training set and test set. The Training Set residuals demonstrate that the model fits the training data well, with minimal systematic errors. The Test Set residuals highlight reasonable generalization but with increased variability, pointing to areas where the model could be refined for better performance on unseen data.

## 5 Discussions

This study successfully developed and validated the COPD Medical Cost Prediction Index (MCPI), an AI-powered tool designed to predict healthcare costs in COPD patients. By incorporating five critical predictors—Age × BMI (interaction term), Number of Comorbidities, Tumor Presence, Acute Exacerbation, and the DOSE Index—the MCPI offers a robust framework for understanding and managing medical expenses in this high-risk population.

The model demonstrated strong predictive performance, explaining 70.3 % of the variance in total healthcare costs in the training set and 36.5 % in the test set. Feature importance analysis identified Tumor Presence, Age, and BMI as the most influential predictors, providing actionable insights for personalized clinical decision-making. Plans to develop a web-based tool for real-time application emphasize the MCPI's potential to enhance clinical workflows and optimize resource allocation.

The MCPI directly supports resource optimization by enabling healthcare providers to identify patients at higher risk for significant medical expenses. This targeted approach facilitates the prioritization of interventions such as intensive monitoring, preventive care, and customized treatment strategies for high-cost individuals. By improving the precision of resource planning, hospitals and healthcare systems can allocate staff, equipment, and budgets more effectively, reducing waste and enhancing cost-efficiency in COPD management.

### 5.1 Strengths

This study's strengths lie in its innovative approach and rigorous methodology. By integrating clinical, demographic, and comorbidity variables, the COPD Medical Cost Prediction Index (MCPI) provides a comprehensive framework for accurately predicting healthcare costs in COPD patients. Unlike traditional models limited to a narrow set of predictors, this study utilized six critical variables—Age × BMI (interaction term), Number of Comorbidities, Tumor Presence, Acute Exacerbation, and the DOSE Index—enabling the model to capture a broad spectrum of cost determinants. The use of Gradient Boosting further enhanced the model's capability to address complex, non-linear relationships among predictors, leading to more robust predictions [36], [37].

A key strength of the study is its robust validation process. The implementation of 5-fold cross-validation ensured the model's generalizability across different data subsets, minimizing overfitting and enhancing reliability. The evaluation framework employed multiple performance metrics, including Mean Squared Error (MSE), Mean Absolute Error (MAE), Mean Absolute Percentage Error (MAPE), and R-squared (R²), providing a comprehensive assessment of the model's accuracy and practical applicability [38], [39].

Another notable strength is the handling of missing data through advanced multiple imputation methods. This approach preserved the dataset's integrity by minimizing information loss and bias while maintaining a sufficient sample size. Consequently, the findings are both valid and applicable to a broader COPD patient population [40], [41], [42].

### 5.2 Limitations

Despite its strengths, this study has several limitations that warrant consideration.

First, the use of a single-center dataset limits the generalizability of the findings to broader COPD populations. Variations in COPD management practices, healthcare cost structures, and resource availability across institutions and regions could impact the model’s performance in different contexts. Future research should focus on validating the MCPI using multicenter datasets that encompass diverse geographic and demographic characteristics.

Second, the exclusion of socioeconomic factors, such as income level, education, and access to healthcare resources, may have constrained the model's ability to fully capture cost determinants. Incorporating these variables in future studies could provide a more comprehensive understanding of healthcare cost drivers in COPD patients.

Third, although multiple imputation techniques were employed to address missing data, residual bias may persist, particularly in cases where data were not missing at random. Future research should explore advanced statistical techniques to further minimize this issue.

Lastly, the relatively small sample size of 396 patients limits the model’s capacity to generalize to diverse populations. Expanding future studies to include larger datasets would improve statistical power and robustness, enabling more detailed subgroup analyses. Such analyses could explore cost drivers specific to different COPD phenotypes or comorbid conditions, thereby enhancing the model's utility in personalized care.

### 5.3 Practical application

The development of a user-friendly web tool is underway to enable real-time implementation of the MCPI in clinical settings. This tool will feature an intuitive interface for clinicians, allowing for quick data input and real-time predictions of medical costs. Designed to integrate seamlessly with electronic health record (EHR) systems, the tool will streamline workflows and minimize manual data entry, enhancing clinical efficiency.

In addition to its primary function, the web tool will incorporate advanced visualization capabilities, offering clinicians graphical insights into cost drivers and the relative contributions of different predictors. For example, bar charts and heatmaps will illustrate the impact of variables such as age, BMI, and comorbidities on predicted costs, facilitating informed decision-making and resource allocation.

The tool will also support scenario analysis, enabling clinicians to modify input variables (e.g., BMI or the number of acute exacerbations) to observe potential changes in cost predictions. This feature will assist in tailoring patient care plans and identifying high-risk individuals who may benefit from targeted interventions.

To ensure accessibility and user engagement, the web tool will include multilingual support and integration with mobile platforms. This will allow healthcare providers in diverse settings to leverage the MCPI for cost prediction, regardless of technological infrastructure or language barriers. Plans for incorporating patient-facing features are also under consideration, providing transparency and empowering patients to understand factors influencing their healthcare costs.

Future iterations of the tool will include machine learning updates, allowing the model to adapt and improve as new data becomes available. Regular feedback from end-users will be collected to refine functionality and ensure the tool meets the evolving needs of clinical practice. The development of a user-friendly web tool is underway to enable real-time implementation of the MCPI in clinical settings. This tool will feature an intuitive interface for clinicians, allowing for quick data input and real-time predictions of medical costs. Designed to integrate seamlessly with electronic health record (EHR) systems, the tool will streamline workflows and minimize manual data entry, enhancing clinical efficiency.

## 6 Conclusions

This study successfully developed and validated the COPD Medical Cost Prediction Index (MCPI), demonstrating strong predictive performance with an R² of 70.3 % for the training set and 36.5 % for the test set. The model exhibited robust accuracy and minimal residual bias, underscoring its potential for clinical application in predicting healthcare costs and optimizing resource allocation for COPD patients.

Despite its strengths, the study's single-center design and the exclusion of socioeconomic variables limit the generalizability of the findings. Future research should focus on validating the MCPI across multicenter datasets to encompass diverse populations and healthcare settings. Additionally, incorporating socioeconomic factors, such as income level, education, and access to healthcare, could enhance the model’s comprehensiveness and precision.

The MCPI represents a significant step forward in leveraging AI-driven tools for personalized care and cost management in COPD, providing a strong foundation for ongoing advancements in this critical area of healthcare.

## Ethical approval and consent to participate

The study was conducted in accordance with the Declaration of Helsinki, and approved by CGMH Review Board Committee (IRB: 201701293B0), and informed consent was waived due to the retrospective design.

## Funding

This research received no external funding

## Author contributions

GH Liu and SF Liu were responsible for the conception and design. CL Li and SF Liu contributed to patient enrolment and data collection. GH Liu, CY Yang and SF Liu analyzed the data. GH Liu, CL Li and SF Liu drafted the manuscript, and CY Yang and SF Liu reviewed the manuscript.

## CRediT authorship contribution statement

**Shih-Feng Liu:** Writing – review & editing, Formal analysis, Data curation, Conceptualization. **Chih-Yuan Yang:** Writing – review & editing, Formal analysis. **Chin-Ling Li:** Writing – original draft, Data curation. **Guan-Heng Liu:** Writing – original draft, Formal analysis, Conceptualization.

## Declaration of Competing Interest

The manuscript is original, has not been published, and is not under consideration elsewhere. All authors have approved the submission, and there are no conflicts of interest to disclose. Should you require any further information or documentation, please do not hesitate to contact me.

## Data Availability

The data supporting this research is available from S.-F.L. C.-L.L.
